# Efficiency of different flows for apneic oxygenation when using high flow nasal oxygen application – a technical simulation

**DOI:** 10.1186/s12871-021-01461-z

**Published:** 2021-10-07

**Authors:** W. A. Wetsch, H. Herff, D. C. Schroeder, D. Sander, B. W. Böttiger, S. R. Finke

**Affiliations:** 1 Department of Anaesthesiology and Critical Care Medicine, University of Cologne, Faculty of Medicine and University Hospital of Cologne, Kerpener Str. 62, 50937 Cologne, Germany; 2 Department of Anesthesiology and Intensive Care, German armed forces Central Hospital of Koblenz, Koblenz, Germany

**Keywords:** Apneic oxygenation, Oropharyngeal oxygenation device, Oxygen desaturation, Simulation

## Abstract

**Background:**

Preoxygenation and application of apneic oxygenation are standard to prevent patients from desaturation e.g. during emergency intubation. The time before desaturation occurs can be prolonged by applying high flow oxygen into the airway. Aim of this study was to scientifically assess the flow that is necessary to avoid nitrogen entering the airway of a manikin model during application of pure oxygen via high flow nasal oxygen.

**Methods:**

We measured oxygen content over a 20-min observation period for each method in a preoxygenated test lung applied to a human manikin, allowing either room air entering the airway in control group, or applying pure oxygen via high flow nasal oxygen at flows of 10, 20, 40, 60 and 80 L/min via nasal cannula in the other groups. Our formal hypothesis was that there would be no difference in oxygen fraction decrease between the groups.

**Results:**

Oxygen content in the test lung dropped from 97 ± 1% at baseline in all groups to 43 ± 1% in the control group (*p* < 0.001 compared to all other groups), to 92 ± 1% in the 10 L/min group, 92 ± 1% in the 20 L/min group, 90 ± 1% in the 40 L/min group, 89 ± 0% in the 60 L/min group and 87 ± 0% in the 80 L/min group. Apart from comparisons 10 l/ min vs. 20 L/min group (*p* = .715) and 10/L/min vs. 40 L/min group (*p* = .018), p was < 0.009 for all other comparisons.

**Conclusions:**

Simulating apneic oxygenation in a preoxygenated manikin connected to a test lung over 20 min by applying high flow nasal oxygen resulted in the highest oxygen content at a flow of 10 L/min; higher flows resulted in slightly decreased oxygen percentages in the test lung.

## Background

Preoxygenation is standard to prevent patients from desaturation e.g. during emergency intubation. The time until desaturation occurs can be prolonged by applying oxygen into the airway and thus providing apneic oxygenation. Established methods to facilitate apneic oxygenation may be application of high flow nasal oxygen via nasal cannulas [1, 2] and some guidelines simply recommend to apply “a bulk full of oxygen”, to maintain apneic oxygenation [3]. From the physiological view, optimum preoxygenation and removal of nitrogen from the lungs are essential for sufficient apneic oxygenation. Any further nitrogen should be prevented from streaming into the upper airway, so only pure oxygen should be allowed to enter the airway in order to maintain apneic oxygenation. However, in a few preliminary studies attaching a human manikin to a test lung, we detected nitrogen entering the upper airway while using nasal cannulas and thus causing a decline of the oxygen content in our test lungs [4–6]. New devices allow high flow nasal oxygenation with a maximum flow of 80 L/min via nasal cannula at 100% oxygen. Using this device should thus facilitate apneic oxygenation, and keep oxygen content of a test lung connected to a human manikin airway constantly at high levels over the time.

Aim of this study was to scientifically assess the optimum flow that is necessary to avoid any nitrogen entering into the airway of a manikin model during application of high flow nasal oxygen (pure oxygen) via nasal cannula. Our formal hypothesis was that there would be no difference in oxygen fraction decrease in a test lung over time at different flow levels.

## Methods

This study is a completely technical simulation using a standardized airway manikin, with no participants. Thus, no ethical approval was required. The trachea of an anatomically correctly shaped male manikin (Laerdal®-Airwaymanagement-Trainer, Laerdal, Stavanger, Norway) was attached to a test lung with a capacity of 2.5 L, simulating the functional residual capacity of a healthy adult male. The test lung was connected at its base to a paramagnetic oximeter that was integrated in a standard anesthesia device (Primus, Dräger, Lübeck, Germany; Accuracy ±(2.5 Vol% + 2.5 rel.)) with a suction rate of 200 mL/min, which is comparable to oxygen consumption during apnea in adults [4–7]. For applying oxygen at high flows to the manikin, we used a standard intensive care respirator (Hamilton-C6, Hamilton Medical AG, Bonaduz, Switzerland) with an Optoflow™ high flow nasal cannula (Optoflow™, Size M, Hamilton Medical AG, Bonaduz, Switzerland) that was adjusted to the manikins’ nostrils.

Before initiation of each experiment, interventional procedures were assigned in random order. We measured in six groups: First a control-group with only room air entering the manikin’s airway and five high flow nasal oxygen groups with oxygen being inflated at flows of 10, 20, 40, 60 and 80 l/min. Five experiments per group were conducted. Before each experiment, the test lung was preoxygenated to 97% oxygen content by use of an oxygen bypass of the mentioned anesthesia machine. Subsequently, the test lung was disconnected from the anesthesia machine but remained connected to the oximeter of the anesthesia device using a standard connection tube for measuring gas samples (Draeger, Lübeck, Germany). We measured the decrease in oxygen percentage in the test lung within a period of 20 min for the above-mentioned settings. The manikins’ mouth remained open in all experiments.

Statistical analysis: Data are reported as mean plus or minus standard deviation. After checking for normality of distribution using Shapiro Wilk test, a one-way analysis of variance (Kruskal Wallis) for repeated measurements was performed to determine overall statistical significance between groups, followed by post hoc Student Newman Keuls test for pair wise multiple comparisons (Sigmaplot 14; Systat, San Jose, CA).

## Results

**Fig. 1:**
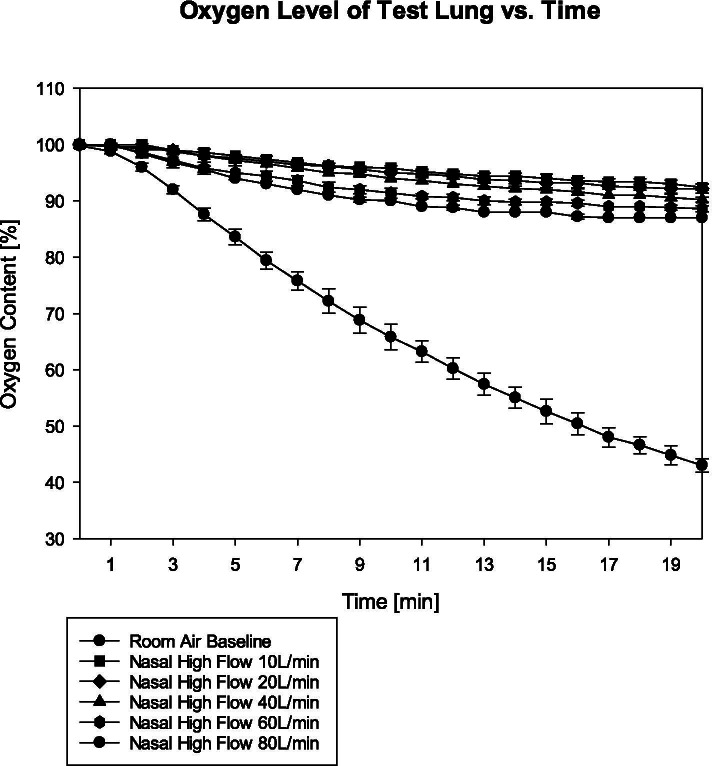
Oxygen levels in the test lung (y-axis) vs. time (x-axis)

A one-way analysis of variance for repeated measurements detected statistical significance between all groups (*p* < 0.001). Oxygen percentage in the test lung dropped from 97 ± 1% at baseline in all groups to 43 ± 1% in the control group (*p* < 0.001 compared to all other groups), to 92 ± 1% in the 10 L/min group, to 92 ± 1% in the 20 L/min group, to 90 ± 1% in the 40 L/min group, to 89 ± 0% in the 60 L/min group and to 87 ± 0% in the 80 L/min group. The course of oxygen percentages for all groups is presented in Fig. 1. Statistical test results are presented in Table 1.

**Table 1:** Test results for comparisons between groups (Student Newman Keuls)

Comparison	Diff of Ranks	q	***P***
High Flow 10 vs Control	114,000	5791	< 0,001
High Flow 10 vs High Flow 80	89,000	5408	0,001
High Flow 10 vs High Flow 60	64,000	4838	0,003
High Flow 10 vs High Flow 40	38,500	3850	0,018
High Flow 10 vs High Flow 20	3500	0,517	0,715
High Flow 20 vs Control	110,500	6714	< 0,001
High Flow 20 vs High Flow 80	85,500	6463	< 0,001
High Flow 20 vs High Flow 60	60,500	6050	< 0,001
High Flow 20 vs High Flow 40	35,000	5170	< 0,001
High Flow 40 vs Control	75,500	5707	< 0,001
High Flow 40 vs High Flow 80	50,500	5050	0,001
High Flow 40 vs High Flow 60	25,500	3767	0,008
High Flow 60 vs Control	50,000	5000	0,001
High Flow 60 vs High Flow 80	25,000	3693	0,009
High Flow 80 vs Control	25,000	3693	0,009

## Discussion

In this model, decrease in oxygen content in a test lung connected to a manikin was lowest at an oxygen flow of 10 L/min via nasal cannula. Higher oxygen flows resulted in slightly decreased oxygen fraction after a 20 min observation period.

Thus, obviously at 100% oxygen, higher flows resulted in higher mixing with nitrogen from ambient air in the upper airway/ open oral cavity. One potential explanation, being discussed before, may be that high and in consequence turbulent flows in the upper airway generate a Bernoulli effects that transports ambient air (and thus nitrogen) into the oral cavity as in a mixing chamber [5, 6]. The higher the flow, the more turbulent they may be, which in consequence increases these Bernoulli effects, resulting in a higher degree of gas mixing in the oral cavity.

Closing the manikins’ mouth might have been protective in this regard. However, we left the manikin mouth deliberately always open due to two reasons: First, to avoid gas trapping at high flow oxygen insufflation. Closing mouth and nose artificially of an unconscious patient while using high flow nasal oxygenation devices may result in gastric inflation with potentially detrimental effects [8, 9]. Second, applying high flow oxygen was intended in this study as a method to extend time until desaturation occurs during intubation efforts. Therefore, an open mouth was mandatory in this study to achieve a realistic scenario, too.

A further aspect explaining more gas mixture using higher gas flows may be effects described by Rittayami et al.: higher gas flows into an airway may result in higher respiratory resistances, which may explain more turbulences and mixture of gases as well. However, all this are theoretical considerations that remain speculative to a certain degree [10].

It is interesting that a gas flow of 10 L/min was most effective in maintaining apneic oxygenation in our model, and that higher gas flows were less effective. As mentioned before gas mixing effects may be one explanation. It is noteworthy that the decrease in oxygen concentration in the test lung was statistically significant, however it should not have been clinically relevant. Even in the “worst group” at a flow of 80 L/min oxygen concentration was 87% after the 20 min observation period, which should be enough to maintain apneic oxygenation. However, these high flows were not necessary in this experiment.

High gas flows may be associated with negative effects. Applying high flow nasal oxygen at higher flow rates results in more aerosol production to the patient’s environment, which can endanger hospital personnel. This has gained much attention especially at the beginning of the COVID-19 pandemic. Fear of virus transmission and subsequently infection caused by infective aerosols [11–13] has even led to the recommendation to avoid high flow nasal oxygen therapy in COVID-19 patients at the beginning of the pandemic. Thus, the lowest possible air flow that maintains adequate oxygen concentrations in a lung during intubation may be considered the optimum from this aspect. There may be other aspects such as better oxygenation due to higher continuous positive airway pressure (CPAP when higher flows are applied using nasal cannulas), which may result in less collapse of alveoli and subsequently better ventilation/perfusion ratio [14]. However, continuous positive airway pressure and subsequently CPAP effects are subject of controversial discussions and especially may vanish when the patient’s mouth is opened to insert a laryngoscope for endotracheal intubation.

There are several limitations to this study. First, this was performed as a manikin study to allow for replicability of the experiments. A manikin study, however, is always a limitation itself, since it cannot completely simulate processes like in a human being. Anatomy, gas exchange, dynamic changes in the upper airways, oxygen consumption and CO2 production during high flow oxygenation are important to determinate the effects of high flow oxygenation on pulmonary oxygenation. Therefore, clinical studies may be mandatory in the future. However, artificially withholding intubation in an operation theatre or even in intensive care medicine to examine the effect of different oxygen flows on oxygen decrease during apneic oxygenation may be difficult to simulate. Thus, to our opinion, for a first assessment of the problem a manikin study may be a useful tool.

One possible scenario to assess these effects in a clinical study is to use different gas flows during rapid sequence induction in ICU settings, where oxygen desaturation sometimes occurs even after a short period of time due to the normal delay between last spontaneous breathing and first artificial ventilation after intubation.

The airway of the manikin was always in patent state. This may be different in humans, where absorption atelectasis or loss of the airway patency which may dramatically shorten the potential off apneic oxygenation to maintain adequate oxygen uptake due to V/Q mismatch [14] or upper airway obstruction. Further, the effect of cardiac output and hemodynamics on oxygen content cannot be assessed in this study, which is another potential limitation. Finally, we were not able to simulate effects of carbon dioxide production and humidification on oxygen content in the test lung, which are effects that have an influence on oxygen content in humans. Animal experiments that may simulate these effects are not applicable due to completely different airway shapes. Further, since apneic oxygenation was detected long ago [15, 16], and applied in multiple studies before [17, 18] a technical simulation was to our opinion an acceptable tool to examine the hypotheses. However, as mentioned before, further clinical studies are mandatory to assess gas flows more precisely that on one side may be beneficial in avoiding arterial oxygen desaturation and on the other side do not result in excessive turbulences and thus gas mixing. To our opinion, any recommendations of such flows cannot be based on technical simulations.

## Conclusion

Simulating apneic oxygenation in a preoxygenated manikin connected to a test lung over 20 min by insufflating pure oxygen via nasal cannula resulted in the highest oxygen content at a flow of 10 l/min; higher flows resulted in slightly decreased oxygen contents in the test lung.

## Data Availability

All data are included in the manuscript. The original datasets analysed during the current study are available from the corresponding author on reasonable request.

## Abbreviations

CPAP: Continuous positive airway pressure
